# eIF4E and eIF4GI have distinct and differential imprints on multiple myeloma's proteome and signaling

**DOI:** 10.18632/oncotarget.3008

**Published:** 2015-01-29

**Authors:** Oshrat Attar-Schneider, Metsada Pasmanik-Chor, Shelly Tartakover-Matalon, Liat Drucker, Michael Lishner

**Affiliations:** ^1^ Oncogenetic Laboratory, Meir Medical Center, Kfar Saba, Israel; ^2^ Department of Internal Medicine, Meir Medical Center, Kfar Saba, Israel; ^3^ Sackler Faculty of Medicine, Tel Aviv University, Tel Aviv, Israel; ^4^ Bioinformatics Unit, G.S.W. Faculty of Life Sciences, Tel Aviv University, Tel Aviv, Israel

**Keywords:** Multiple Myeloma, Translation initiation, eIF4GI, eIF4E, Bioinformatics

## Abstract

Accumulating data indicate translation plays a role in cancer biology, particularly its rate limiting stage of initiation. Despite this evolving recognition, the function and importance of specific translation initiation factors is unresolved. The eukaryotic translation initiation complex eIF4F consists of eIF4E and eIF4G at a 1:1 ratio. Although it is expected that they display interdependent functions, several publications suggest independent mechanisms.

This study is the first to directly assess the relative contribution of eIF4F components to the expressed cellular proteome, transcription factors, microRNAs, and phenotype in a malignancy known for extensive protein synthesis-multiple myeloma (MM). Previously, we have shown that eIF4E/eIF4GI attenuation (siRNA/Avastin) deleteriously affected MM cells' fate and reduced levels of eIF4E/eIF4GI established targets. Here, we demonstrated that eIF4E/eIF4GI indeed have individual influences on cell proteome. We used an objective, high throughput assay of mRNA microarrays to examine the significance of eIF4E/eIF4GI silencing to several cellular facets such as transcription factors, microRNAs and phenotype. We showed different imprints for eIF4E and eIF4GI in all assayed aspects. These results promote our understanding of the relative contribution and importance of eIF4E and eIF4GI to the malignant phenotype and shed light on their function in eIF4F translation initiation complex.

## INTRODUCTION

Control of protein translation is an important strategy by which eukaryotic cells regulate gene expression. Thus, it is hardly surprising that deregulation of translation has been linked to various human malignancies, multiple myeloma (MM) included [1]. Targeting translation is particularly attractive in the incurable malignancy of MM because of the cells' extensive protein synthesis and secretion, a unique and distinguishing feature [2]. Translation initiation rigorously regulated, is rate-limiting to protein synthesis, and frequently deregulated in cancer, including MM [3–8]. The process is dependent on recruitment of eukaryotic initiation complex eIF4F, composed of cap binding eIF4E, scaffolding protein eIF4G, and RNA helicase eIF4A. eIF4E and eIF4G specifically, were proven to be critical for translational control, inactivated by stress, activated by growth promoting signals, and often elevated in cancer [5]. It is established that eIF4E is rate limiting to 5′ cap dependent translation typical of 90% of cellular proteins [4], and that eIF4G (I, II) is a key initiator of eIF4F-complex assembly [9]. Despite the universal usage of both factors in the eIF4F complex, accumulated data suggests that some protein targets characterized with complex 5′UTRs rely to a greater extent on eIF4E for translation and presumably its association to the 5′ cap [10], whereas other proteins are translated in a correlation with eIF4G function [11]. Of note, many of the recognized target proteins promote cancerous processes and drug resistance [1]. In previous reports we have shown that attenuation of eIF4E and eIF4GI with the VEGF inhibitor, Bevacizumab [12] resulted in decreased levels of the factors and their established targets. Moreover, in two additional reports we knocked down the factors (siRNA, 40–60% silencing) and witnessed, again, reduced levels of the same specific established targets with no reciprocal influence [13, 14]. Our results support previous data that inhibition of both translation initiation factors (separately) did not greatly affect global translation yet resulted in depletion of specific proteins important to the malignant process [9, 11, 12]. Our findings are in concordance with the suggestion of Cenci et al. [15] that eIF4E and eIF4GI dictate not only rate of protein synthesis but its quality as well. This observation calls attention to the unresolved questions regarding translation initiation and the on-going debate regarding its underlying mechanisms, with specific emphasis on cap-dependent versus cap-independent translation. Borrowing from viral systems, cap-independent translation may be implemented by utilization of internal ribosome entry sites (IRES) and eIF4G un-complexed in the traditional eIF4F [16]. Additional evidence for IRES-dependent translation comes from synthetic models, which suffer from fundamental disadvantages [17]. The eukaryotic translation initiation complex eIF4F is composed of eIF4E and eIF4G at a 1:1 ratio. Thus, it is expected that eIF4E and eIF4G display interdependence. Disparities in the respective utilization of eIF4E and eIF4G may support eIF4F independent mechanisms. While the studies on these translation initiation factors were conducted on quite a few models altogether they have not been systematically and simultaneously explored in a single setting. Furthermore, reviewing established data on translation imitation factors in MM reveals paucity of information.

Thus, this study concentrated on a particular cancer model and studied the role of eIF4E and eIF4GI in the design of the cells' proteome. We used an unbiased, high throughput system to evaluate the individual importance of eIF4E and eIF4GI levels in MM. We used models of eIF4E or eIF4GI knocked down (KD) MM cell line RPMI 8226 and profiled their respective translated transcription factors (TF), often tumor suppressors or oncogenes. Furthermore, we assessed the KDs' microRNAs repertoires and cells' phenotype. Significant differences were observed between eIF4E and eIF4GI knockdown imprints.

## RESULTS

### Our experimental model

In our study we used the well recognized and authenticated RPMI 8226 MM cell line. Employing siRNA we knocked down the expression of eIF4E or eIF4GI or delivered negative siRNA as control [13, 14]. Target silencing was validated 24 h post-transfection at the transcript level (50%↓ for si-eIF4E and 60%↓ for si-eIF4G, *p < 0.01*) and 48 h post-transfection at the protein level (40%↓ for si-eIF4E and 60%↓ for si-eIF4G) [13, 14]. The knock down was deliberately of limited extent so as to avoid dramatic cell damage due to protein synthesis shutdown that would mask the nuances of the relative translation initiation factors. Total RNA extracted from 120 h post transfection cells was applied to whole genome Affymetrix microarray chips at the Bioinformatics Unit, Tel-Aviv University. Batch effect of the 2 separate experiments was removed using Partek Genomics suite based on principal component analysis (PCA) demonstrating that the variations between the duplicates were not prominent. Results are presented in the following chapters. Of note, all findings were validated in 5 separate experimental replicates.

### Distinct and significant differences exist between eIF4E/eIF4GI translation initiation modes

**Figure 1:**
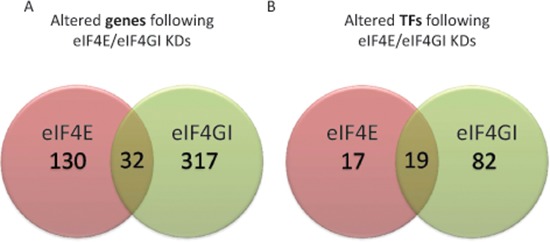
RPMI 8226 eIF4E/eIF4GI KDs demonstrated distinct dissimilarities in transcribed genes' repertoires and TFs RPMI 8226 cells were transfected with negative or anti eIF4E siRNA or anti eIF4GI siRNA. Total RNA was extracted 120 h post transfection and applied to whole genome Affymetrix microarray chips. Significantly altered gene lists were generated in comparison to negative control (*p < 0.05* and 1.25 fold expression difference). Genelists were compared using Venny and newly produced lists of common and exclusive components of eIF4E/eIF4GI KDs (Venn diagram) were analyzed: **(A)** whole genome **(B)** panels of altered TFs deduced from whole genome data using bioinformatics tools (Webgestalt, ToppGene).

In our preceding studies we showed dependence of specific known targets on eIF4E/eIF4GI [12–14]. In those studies we used a KD model of eIF4E or eIF4GI in MM cell lines and demonstrated reduced targets' expression 96 hours post-transfection without reciprocal effect [13, 14]. Here, we obtained lists of genes with the Affymetrix microarrays depicting changes in expression upon eIF4E/eIF4GI KD 120 hours post-transfection. Using the nonparametric Kolmogorov-Smirnov we concluded that the data distribution is not normal. Hence, we applied the Wilcoxon signed ranks test and determined significant differences between the KDs' gene lists output (*p*v* = 0.00048*). These results suggested that there is a distinct dissimilarity in the effects of eIF4E and eIF4GI KDs on the transcribed genes' repertoire (Figure 1A), as most of the genes were unique to each treatment and only a small fraction was common. Differences between eIF4E and eIF4GI KDs were further characterized employing various bioinformatics tools (material and methods).

### eIF4E/eIF4GI KDs affected different transcription factors important to the tumorigenic phenotype of MM cells

Previous data from our studies [13, 14] and those of others [18–20] demonstrated a considerable presence of transcription factors (TF) among the recognized eIF4E/eIF4GI targets. Those TFs have established importance to MM pathogenesis and progression [18–21]. In the present work we used bioinformatics predictions to uncover additional TFs whose expression was changed upon eIF4E/eIF4GI KD (based on the identification of the gene expression of TFs targets in the Affymetrix chips). Analyses with Webgestalt and ToppGene produced different panels of TFs for eIF4E and eIF4GI KDs (Table 1, Figure 1B) that, interestingly, included at least one TF we have already assayed based on published data [13, 14]. The list of altered TFs included 36 candidates for eIF4E and 101 candidates for eIF4GI of which we selected for further analyses the TFs with the highest *p*-value scores (*0.0046 < p < 1.40e-08*) (for eIF4E: ETS2, SP1, AP1, NFkB; for eIF4GI: FOXO4, LEF1, SREBP1, ERα, HIF1α). Bioinformatics predictions were validated at the mRNA level by qPCR and at the protein level by immunoblotting (*n* = 5). Indeed, all assayed bioinformatics observations were demonstrated in all 5 experimental repeats of the KD experiments. Moreover, no reciprocal effect was demonstrated when we tested the TF's target genes of eIF4E in the eIF4GI KD cells and *vice-versa*. Table 1 summarizes the selected and validated TF's, their targets, change rates, importance to the cells' fate, and relevance to the malignant progression.

**Table 1:** eIF4E/eIF4GI's TFs and their targets genes

eIF4E's TFs	Relevance to the malignant phenotype	TFs' targets genes	Validated TFs' targets genes	% Change^*^
ETS2 *(p = 3.43e-06)*	Nuclear proto-oncogenes that correlated with cell proliferation, differentiation, and apoptosis [45].	AGPAT1, DOCK4, ARAP1, SERPINI1, PTPRC, EGR1, LSP1	↓AGPAT1	30%↓
			↑DOCK4	63%↑
SP1 *(p = 0.0006)*	Plays a major role in regulating expression of cell differentiation, cell cycle, and apoptosis-related genes affecting cellular growth [46].	AGPAT1, DOCK4, ARAP1, SERPINI1, eIF4B,	↓ARAP1	40%↓
			↓SERPINI1	50%↓
			↑IL23A	52%↑
AP1 *(p = 0.0006)*	Regulates a wide range of cellular processes, including cell proliferation, death, survival and differentiation [47].	IL23A, CRYGS	↓PTPRC	60%↓
			↓EGR1	40%↓
NFkB *(p = 0.0163)*	One of the most important pathways in MM for the cells' survival and proliferation. Mutations involving the NFkB pathway are present in at least 17% of MM tumors and 40% of MM cell lines [48].	AGPAT1, IL23A, TRIB2	↓LSP1	25%↓
			↑eIF4B	55%↑
			↓CRYGS	45%↓

eIF4GI's TFs	Relevance to the malignant phenotype	TFs' targets genes	Validated TFs' targets genes	% Change^*^
FOXO4 *(p = 7.35e-07)*	Regulation of cell cycle, apoptosis, survival, and response to oxidative stress [49]	FBXO32, GAB2, HOXB9, PIM1, BCL2, SESN2	↓FBXO32	60%↓
			↓GAB2	60%↓
LEF1 *(p = 1.32e-05)*	Pro-survival factor. Mediator of the Wnt signaling pathway that regulate genes that control survival and the cell cycle [50].	FBXO32, GAB2, HOXB9, PIM1, ASS1	↓HOXB9	50%↓
			↓PIM1	40%↓
SREBP1 *(p = 0.0005)*	Regulation of fatty acid and phospholipid synthesis. Recent study demonstrated the connection between SREBP1 regulation of lipid synthesis to cell survival and tumor growth [51]	FBXO32, GAB2, DDIT3	↓BCL2	60%↓
			↓SESN2	55%↓
ERα *(p = 0.0017)*	Modulation of ERα in MM cell lines reduced viability, induced caspase-dependent apoptosis, and induces myeloma cell cycle arrest and apoptosis.	HOXB9, PPM1E	↓ASS1	55%↓
			↓DDIT3(CHOP)	45%↓
HIF1α *(p = 0.0074)*	Up-regulated in MM cells [52]. key transcription factor regulating the expression of genes involved in glycolysis and angiogenesis [53].	PIM1, BCL2	↓PPM1E	65%↓

* All results are significantly changed in eIF4E/eIG4GI KDs compared to negative control si-RNA (*p < 0.05*).

(TF = Transcription Factor). (__)Underline depicts TFs validated at the protein level (Supplementary Figure S1).

### eIF4E/eIF4GI KD affected microRNA

**Figure 2:**
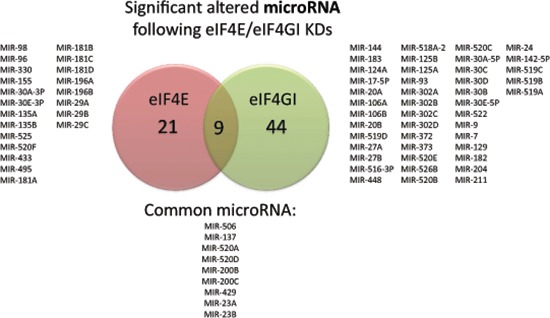
Effect of eIF4E/eIF4GI KDs on predicted microRNA repertoire in RPMI 8226 cells RPMI 8226 cells were transfected with negative/anti eIF4E siRNA/anti eIF4GI siRNA. Total RNA was extracted 120 h post transfection and applied to Affymetrix microarray chips. Venn diagram showed common and uncommon predicted microRNAs analyzed and deduced with bioinformatics tools (Webgestalt, Toppgene) in eIF4E/eIF4GI KDs. The microRNAs' symbols are indicated. All indicated microRNAs were significant in bioinformatics analysis (*p < 0.05*).

The common involvement of microRNAs and translation initiation factors in proteome regulation and MM progression [22] led us to hypothesize that there may be a regulatory link as well. Thus, we examined microRNAs' expression in the translation initiation factors silenced models. Affymetrix gene expression output was analyzed with bioinformatics tools (Webgestalt, ToppGene) for predicted microRNAs. Results demonstrated that the KDs significantly (*p < 0.05*) affected microRNAs repertoire with lists of 30–50 possible microRNAs in eIF4E/eIF4GI KD models (Figure 2). Of note, the eIF4E dependent microRNAs were dissimilar to the eIF4GI dependent microRNAs (Figure 2) in term of specific MiRs. Suggested microRNAs with previous publication relating them to MM and/or the malignant niche are presented in Table 2. Selected microRNAs expressions were validated by qPCR in 5 KD experiments (underlined in Table 2). To the best of our knowledge, this is the first analyses of the effect of translation factors on microRNAs and not *vice versa*.

**Table 2:** MicroRNAs analysis of eIF4E/eIF4GI KD

Silenced translation initiation factor	Modulated miRNAs	General miRNAs known function	Specific microRNAs connection to MM	ref	Validation
eIF4E	MIR-29B *(p = 0.0420)*	Apoptosis	miR-29b sensitizes multiple myeloma cells to bortezomib-induced apoptosis	[54]	40%↑ *(p = 0.014)*
		Osteoblast differentiation	miR-29b contribute to positive regulation of osteoblast differentiation in MSC	[55]	
	MIR-155 *(p = 0.0066)*	Cell survival	Significantly down-regulated in MM cells	[56]	NT
	MIR-135B *(p = 0.0066)*	Genetic subtype and survivalOsteogenic differentiation	Significantly up-regulated in MMImpaired osteogenic differentiation of MSC in MM	[57]	NT
	MIR-96 *(p = 0.0016)*	Prognostic marker in hematological malignancy	Significantly down-regulated in hematological malignancy	[58]	60%↑ *(p = 0.04)*
eIF4GI	MIR-20A *(p = 0.0034)*	Angiogenesis	Significantly up-regulated in MM	[56]	NT
	MIR-17-5P *(p = 0.0034)*	Hematopoietic differentiation	Significantly up-regulated in MM	[56]	NT
	MIR-27A *(p = 0.0034)*	Differentiation of MSC	miR-27 is an activator of the Wnt signaling pathway.	[59]	60%↑ *(p = 0.006)*
	MIR-211 *(p = 0.0180)*	ER stress	Regulates chop expression in a PERK-dependent manner	[60]	50%↑ *(p = 0.036)*
eIF4E and eIF4GI	MIR-23A *(p = 0.0362)*	Tumor suppressor	miR-23 is inhibiting cellular proliferation by targeting CREB1 in MM		E: 210%↑ *(p = 0.025)*
			Significantly up-regulated in MM	[56]	G: 60%↑ *(p = 0.049)*

(*) All results are significant (*p < 0.05*). (NT) depicts bioinformatics results not validated by qPCR. (__) Underline depicts microRNAs validated with qPCR.

### Functional Gene Ontology (GO) analysis showed a significant phenotypic difference between eIF4E/eIF4GI KDs'

**Figure 3:**
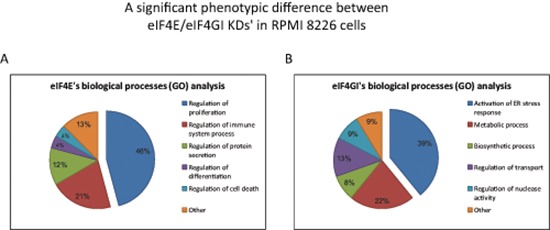
Functional Gene Ontology (GO) analysis showed a significant phenotypic difference between eIF4E and eIF4GI KDs' in RPMI 8226 cells Cells were transfected with negative/anti eIF4E siRNA/anti eIF4GI siRNA. Total RNA was extracted 120 h post transfection and applied to whole genome Affymetrix microarray chips. The microarray data was assessed for biological process function using GO enrichment tools (Webgestalt, Toppgene). Select enriched functions were chosen for demonstration for eIF4E KD data **(A)** and eIF4GI KD data **(B)** The percentage of altered genes involved in the various biological processes is indicated by numbers in the pie chart. All indicated processes were significant in bioinformatics analysis (*p < 0.05*).

Enriched functional analysis of the Affymetrix data with bioinformatics tools (Webgestalt, Toppgene) was implemented and only predictions that were significant with both tools were considered valid. Distinct changes in biological processes of several major categories for each knockdown model (Figure 3) were observed. While the eIF4E KD outlined changes in proliferation, immune response, and protein secretion, the eIF4GI KD showed enrichment in activation of stress response (ER and autophagy), metabolic process, response to stimulus, and regulation of transport and nuclease activity. It can be concluded that not only eIF4E and eIF4GI each have a particular gene-set but these have significantly different functional assignment from that of the other.

We chose to concentrate on the most significantly affected category of each factor i.e. proliferation in the eIF4E KD and ER-stress in the eIF4GI KD. Of note, none of the highlighted biological processes in the eIF4E KD were related to ER-stress and none of the significant biological processes in the eIF4GI KD were related to proliferation. A more detailed list of the enriched GO results in the selected categories is provided in Table 3. In order to validate the above processes and to confirm their dependence on the respective translation initiation factors we conducted several experiments detailed in the next sections.

**Table 3:** eIF4E/eIF4GI's KD GO analysis-Biological processes

siRNA-eIF4E		
Proliferation	WBG	Topp
Regulation of lymphocyte proliferation	0.0016	6.17E-04
Regulation of mononuclear cell proliferation	0.0017	6.46E-04
Regulation of B cell proliferation	0.002	4.03E-04
Regulation of leukocyte proliferation	0.0021	8.13E-04

siRNA-eIF4GI		
ER Stress response	WBG	Topp
Response to endoplasmic reticulum stress	0.0002	9.90E-04
Cellular response to unfolded protein	0.0005	6.41E-04
Endoplasmic reticulum unfolded protein response	0.0005	5.19E-04
Cellular response to topologically incorrect protein	0.0007	9.15E-04
ER-nucleus signaling pathway	0.0009	1.02E-03
Activation of signaling protein activity involved in unfolded protein response	0.0014	1.85E-03
Regulation of autophagy	0.038	2.96E-02

### Prolonged inhibition of eIF4E caused decreased cell proliferation

**Figure 4:**
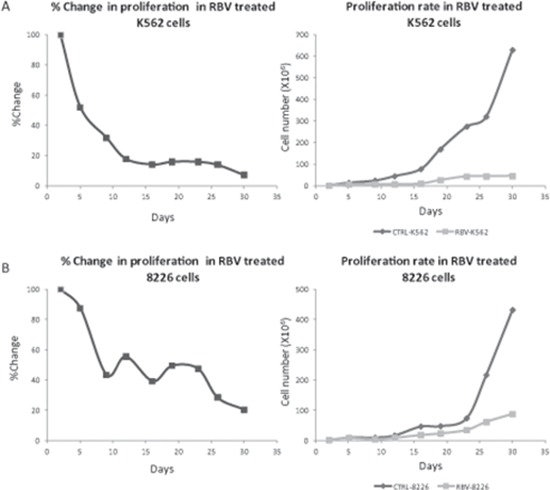
Long term inhibition of eIF4E with RBV attenuated cell proliferation MM cell line RPMI 8226 **(A)** and CML cell line K562 **(B)** were cultured in the presence of RBV (5 μM and 7 μM, respectively) for 30 days. Cells counts taken every 3–4 days (trypan blue) were compared to untreated cells. Results are presented as ratio of treated/untreated cells (left) and as an absolute cell number (right). At least three separate experiments were conducted for each time point. All results are statistically significant (*p < 0.05*).

In contrast to our GO analyses findings our previous assessments of short term (120 hours) eIF4E inhibition (KD, bevacizumab, Ribavirin) [12, 14] did not affect cell proliferation. Thus, we wondered if a long term eIF4E diminution is necessary to realize its significance to the MM cells proliferation. We used Ribavirin (RBV-competitive inhibitor of eIF4E [23]) instead of siRNA thereby enabling the inhibition of eIF4E activity for an extended period of time. RBV's IC50 in RPMI 8226 cells was determined using viability assay as 5 μM [14]. Next, RPMI 8226 MM cells were cultured with regular media supplemented with RBV (5 μM) for 30 days. Media was replaced twice weekly and cells were counted using trypan blue (live/dead cells) every 3–4 days. Indeed, the RBV treatment caused a significant inhibition of the cell proliferation compared to untreated cells (Figure 4A). Ratio analysis of the RBV treated cells compared to untreated control cells showed reduction of 13% in RPMI 8226 cell counts after five days, 50–60% decrease after 9–26 days and an 80% decrease in cell counts after 30 days (*p < 0.05*). Interestingly, no changes in the cells' death rates were evident (data not shown). After establishing that long term eIF4E inhibition affected RPMI 8226 proliferation we wondered whether this affect would be more prominent in highly proliferating cells. For this purpose we used the CML cell line K562 that proliferates substantially faster than MM cell lines (12 h Vs. 56 h) [24, 25]. We determined RBV's IC50 in K562 according to the cell's viability at 7 μM at 24 h. Employing the same experimental design we used with RPMI 8226 we exposed K562 cells to RBV (7 μM) for 30 days, replaced media and drug twice weekly and counted cells. Again, we witnessed a significant reduction in cell counts, yet the response was earlier (5 days versus 9 days) and more profound with 90% reduction in cell counts after 30 days (versus 80%, *p < 0.05*) (Figure 4B). Here too, no changes in the cells death rates were evident (data not shown).

Altogether, these results suggest that eIF4E is critical to cell proliferation and that cells characterized with greater proliferation rates are more susceptible to its inhibition.

### Inhibition of eIF4GI increased ER-stress and UPR signaling

Next, we wanted to validate the GO enrichment analysis that eIF4GI inhibition activated pathways related to ER-stress, UPR and autophagy. Thus, we compared levels of various markers of UPR and autophagy signaling pathways in eIF4GI KD model to negative control.

**Figure 5:**
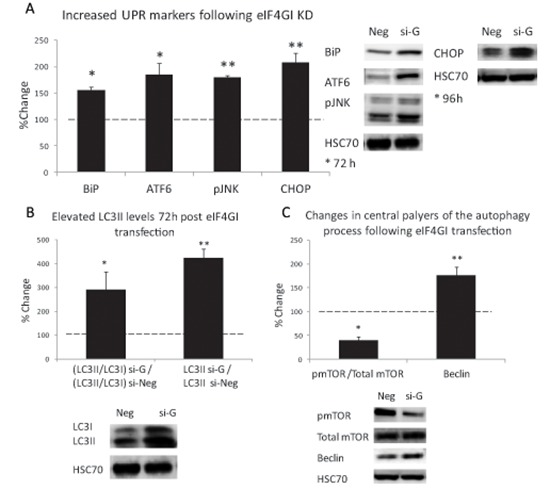
eIF4GI KD Increased ER-stress and UPR signaling and enhanced autophagy response in RPMI 8226 MM cell line RPMI 8226 cell line was transfected with negative (Neg) or eIF4GI siRNA (si-G) and assayed for: **(A)** UPR signaling pathways; **(B)** autophagic marker LC3-II (72 h post transfection); **(C)** autophagy inhibitor-pmTOR and a component of the autophagic machinery-Beclin1 (48 h and 96 h post transfection respectively). Representative immunoblots and graphic presentations (mean ± SE) are presented 72/96 hours post-transfection. HSC-70 served as a loading control. All assayed parameters measured in transfected cells were compared to negative control transfected cells and expressed as change percent. Statistically significant differences (*; *p* < 0.05, **; *p* < 0.01) are depicted.

We assessed levels of the ER-stress sensor, GRP78/BiP, ATF6, the IRE1 pathway component pJNK (Thr183/Tyr185), and the PERK pathway component GADD153/CHOP [26]. Significant elevations of BiP and the three UPR arms were determined in eIF4GI KD RPMI 8226 cells in comparison to negative control (BiP 56%↑; ATF6 85%↑; pJNK 80%↑ 72 h post-transfection, *p < 0.05*; CHOP 107%↑ 96 h post-transfection, *p < 0.01*) (Figure 5A). These results demonstrate a profound UPR activation thereby substantiating increased ER-stress in the eIF4GI KD MM cell line RPMI 8226. Next, we assayed the expression levels of autophagy markers. We observed a significant elevation in the proportion of LC3II (vs LC3I) in eIF4GI KD RPMI 8226 cells compared to negative control cells (Figure 5B) (190%↑, *p < 0.05*). Our results also displayed significant increases in absolute LC3II levels (not relative to LC3I) expressed in eIF4GI KD model compared with negative control cells (320%↑, *p < 0.01*), an analysis method suggested to be more reliable for determining autophagy [27]. For additional validation of autophagic modulation, we examined levels of the established inhibitor of autophagy mTOR, a recognized target in MM therapeutics [28]. Assessment of phosphorylated and total mTOR levels in si-eIF4GI transfected RPMI 8226 cells displayed decreased levels of active mTOR compared with negative control (40%↓, 48 h post-transfection, *p < 0.01*), which is in sync with autophagic activation (Figure 5C). Finally, we assessed cellular levels of Beclin1, an established component of the autophagic machinery [29]. Again, we witnessed elevated Beclin1 levels in RPMI 8226 cells 96 hours post anti-eIF4GI si-RNA transfection (76%↑, *p < 0.05*) (Figure 5C) compared to negative control.

As we detailed in previous publication, in our transfection experiments the anti-eIF4GI siRNA did not have major influence on cell death levels [13]. Thus, we wanted to examine if the cells were using autophagy in order to save themselves from the stress induced by eIF4GI inhibition. For this purpose we treated RPMI 8226 eIF4GI KD cells with the established inhibitor of autophagy 3-methyladenine (3MA-inhibiting autophagosome formation) and re-assessed cell death. Indeed, 96 hours post-transfection we observed 43% cell death in cells co-treated with 3MA and siRNA for eIF4GI in comparison to 23% death in cells treated only with anti-eIF4GI siRNA (*p < 0.05*). Our findings indicate that eIF4GI levels in MM are pertinent to the maintenance of the cells equilibrium. Forcefully depleting the translation initiation factor's levels causes the cells to enter a stressful state and initiate protective measures.

### Assay of biological relevance of the Timlip in additional experimental models

In order to test the validity of our Timlip for eIF4E and eIF4GI we assayed the expression of the designated and established targets in MM cell lines treated with an eIF4E inhibitor (RBV) (RPMI-8226, CAG) and an inhibitor of the factors' association (4EGI) (RPMI 8226, U266, ARK, ARP1) as described in our previous publication [12–14]. Results confirmed the dependence of the listed targets on each/both translation initiation factors, respectively (Table 4).

**Table 4:** 4EGI and Ribavirin (RBV) effect on eIF4E/eIF4GI targets

eIF4E and targets			eIF4GI and targets			eIF4E/eIF4GI's common targets		
	4EGI	RBV		4EGI	RBV		4EGI	RBV
eIF4E	↓	ND	eIF4GI	↓	ND	cMyc	↓	↓
NFkB	↓	↓	SMAD5	↓	=	HIF1α	↓	↓
CyclinD	↓	↓	ERα	↓	=			

* All the values in the table indicates changes in the protein level between 20%–60% (*p < 0.05*) in MM cell lines (RBV: RPMI 8226, CAG. 4EGI: RPMI 8226, U266, ARK, ARP-1) as described in our previous publication [12–14]. ND: not determined; = : unchanged

**Figure 6:**
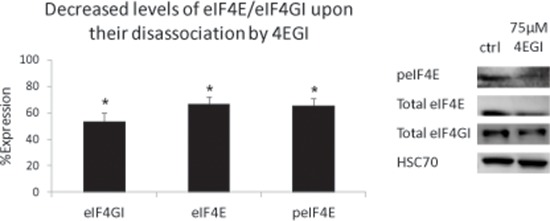
Decreased levels of eIF4E/eIF4GI upon their disassociation by 4EGI MM cell lines were treated with 4EGI (75 μM, 72 h) and immunoblotted for eIF4GI and peIF4E/Total eIF4E. Representative immunoblots (representative bands, right) and graphic presentations (Average value of MM cell lines, left, mean ± SE) are presented. HSC-70 served as a loading control. Statistically significant differences (*; *p* < 0.05, **; *p* < 0.01) are depicted.

Surprisingly, we also observed a decrease in total eIF4E and eIF4GI levels upon their disassociation by 4EGI (Figure 6). To the best of our knowledge, this is a novel observation. Future studies are necessary to determine if the factors undergo increased degradation upon disassociation and whether the degradation is proteosome dependent.

Additional studies conducted in our laboratory applied our Timlip imprints to several experimental systems in order to identify eIF4E/eIF4GI dependency following different manipulations. Thus, we examined the effect of VEGF inhibition (with bevacizumab (BVZ)) on eIF4E/eIF4GI and their targets [12, 13]. Alignment of Bevacizumab's results with the Timlip imprint revealed that Bevacizumab's effect is not compatible with the inhibition of one of the translation initiation factors but with the inhibition of both. These results suggest that VEGF signaling is mediated via both eIF4E and eIF4GI and as far as we know this is a new understanding (Table 5).

**Table 5:** Comparison of the changes of eIF4E/eIF4GI and their targets' levels following siRNA or Bevacizumab treatments

eIF4E			eIF4GI			eIF4E & eIF4GI		
	KD	BVZ		KD	BVZ		KD	BVZ
eIF4E	40%↓	35%↓	eIF4GI	60%↓	30%↓	cMyc	30%↓	23%↓
NFkB	20%↓	50%↓	SMAD5	30%↓	25%↓	HIF1α	35%↓	50%↓
CyclinD	40%↓	34%↓	ERα	30%↓	20%↓			
MMP9	30%↓	35%↓						

* The values in the table indicates changes in the protein level (*p < 0.05*).

Finally, we also examined the Timlip data on non-myeloma systems such as lung cancer cells and bone marrow mesenchymal stem cells and observed similar trends (data not published).

## DISCUSSION

In the current work we explored whether eIF4E and eIF4GI translation initiation factors each have a unique and distinguishable influence on MM cells. Our findings substantiate that indeed profound differences exist in translation initiation factors' impact in our research model. The diverse imprints for eIF4E and eIF4GI in MM cells are evident at multiple expression levels, beginning with the transcription factors and ending at the cells' phenotype. Importantly, our findings support a distinct role for eIF4E in cell proliferation whereas eIF4GI has a significant role in the cells stress responses.

Our observations regarding the particular role of eIF4E are compatible with previous publications [30, 31]. Genome wide techniques for studying translation have shown that constitutive or induced [31] overexpression of eIF4E affects the expression of proteins that are strongly implicated in cell growth, proliferation and survival. On the contrary, reports of eIF4GI in stress responses were limited to situations of viral infections and utilization of the IRES [32].

“Transcriptome studies” investigate the differences in the steady-state abundance of mRNA under different conditions. However, studies have shown that there is often a poor correlation between the abundance of the mRNA in comparison to the protein, which can be explain by translational or post-translational mechanisms that control the proteome profile [33]. Here, we took advantage of a technique developed in the Post-Genomic era that allowed discovery of as yet undetermined associations, which allowed us to classify mRNAs into groups based upon their dependence on specific factors in the eIF4F complex [34]. In order to assemble the imprint lists we silenced eIF4E and eIF4GI separately and applied an objective, high throughput assay which allowed us to assemble a distinguishing imprint list for each factor, i.e. a particular “Timlip”: Translation Initiation Mode Litmus Paper as detailed in Table 6. Our Timlip lists addressed the following predicted facets: transcription factors, microRNAs and phenotype. Validated findings are indicated as well.

**Table 6:** “Translation initiation mode litmus paper” - Timlip^*^

	eIF4E's Timlip				eIF4GI's Timlip				Identification method
Established targets (not TF)	Cyclin D MMP9								Western blot
Established targets-TFs	NFkB HIF1α c-Myc				SMAD5 ERα HIF1α c-Myc				Western blot
TFs from Microarray analyses	ETS2	SP1	AP1	NFkB	FOXO4	LEF1	SREBP1	ERα	Western blot
TFs' targets genes	AGPAT1 DOCK4 ARAP1 SERPINI1 PTPRC EGR1 LSP1	AGPAT1 DOCK4 ARAP1 SERPINI1 eIF4B	IL23A CRYGS	AGPAT1 IL23A TRIB2	FBXO32 GAB2 HOXB9 PIM1 BCL2 SESN2	FBXO32 GAB2 HOXB9 PIM1 ASS1	FBXO32 GAB2 DDIT3	HOXB9 PPM1E	qPCR
Modulated microRNAs	MIR-29B MIR-96 MIR-155 MIR-135B				MIR-27A MIR-211 MIR-20A MIR-17-5P				qPCR
Effect on phenotype	Attenuated proliferation				ER Stress response: Unfolded protein response; Autophagy				Western blot (UPR signals) Cell count

* Grey font color indicates predicted but not validated Timlip component versus

**Black** font colored components that are both predicted and validated.

Our findings support accumulating data that indicate that eIF4E and eIF4GI may have individual influence on the cells' proteome [12–14, 31] despite their recognized association and function in the eIF4F complex [35]. Most importantly, our findings support the emerging understanding that the conventional paradigm of cap binding translation should be revised [34]. Moreover, this study puts forward the implication of the eIF4E/eIF4GI translation initiation modes to the content of the “translatomes” (genome wide pools of translated mRNA) in MM cells.

In the clinical aspect, we suggest that Timlip screening may facilitate design of optimized drug combinations that overcome drug resistance. Since drug resistance remains a major clinical challenge for cancer treatment it is becoming necessary to use a high throughput system to screen predictive markers for optimized drug combinations. Several publications have demonstrated that enhanced UPR stress response or elevated levels of cell proliferation can achieve drug resistance in cancer cells [36, 37]. Our knockdown results have clearly established eIF4E/eIF4GI promote phenotypic responses that could assist the cells to acquire resistance. Further study is needed to establish the interdependence of drug resistance and eIF4E/eIF4GI levels. Positive results will underscore the advantage of applying the Timlip screening to cancer cells with drug resistance and offer a new approach to drug combination design that uses a high throughput method to screen cellular changes in response to specific chemotherapy.

## MATERIALS AND METHODS

### Cell lines

The MM cell line RPMI 8226 and AML cell line K562 (ATCC, Manassas, VA, USA) were cultured in RPMI 1640 as described previously [38] (Biological Industries, Kibbutz Beit-Haemek, Israel).

### Immunoblotting

Cells were lysed, proteins were extracted and western blot was preformed as described elsewhere [38]. The following proteins were detected with Rabbit/Mouse anti-human: eIF4E, eIF4GI, pmTOR(Ser2448)/total mTOR c-Myc, FOXO4 (Cell Signaling Technology, Danvers, MA, USA), SMAD5, HSC70 (Epitomics, Burlingame, CA, USA), ERα (Millipore Billerica, MA, USA), HIF1α, NFkB (Santa-Cruz, CA, USA) and LC3/LC3II (Sigma). Bound antibodies were visualized using peroxidase-conjugated secondary goat anti rabbit or mouse antibody (Jackson ImmunoResearch Laboratories, West Grove, PA, USA), followed by enhanced chemiluminescence (ECL) detection (Millipore). Products were visualized with LAS3000 Imager (Fujifilm, Greenwood, SC, USA). Integrated optical densities of the immunoreactive protein bands were measured as arbitrary units employing Multi Gauge software v3 (Fujifilm).

### siRNA transfection

Validated Alexa-labeled AllStars negative control and anti eIF4E (20 pmol) and combination of five different sequences anti eIF4G (20 pmol: 4 pmol each, 4 designed by Qiagen and 1 published previously [39]) (Qiagen) were delivered into RPMI 8226 MM cell line using lipofectaime2000 (Invitorgene, Carlsbad, CA, USA). Fluorescence (≥10,000 events/analysis) was analyzed by flow cytometery (Navios, Beckman Coulter, USA) and determined in 96% of the cells (transfection efficiency). Silencing of eIF4E/eIF4G was detected at the RNA level by qPCR and at the protein level by western blot 24 h, 48 h, 72 h, 96 h, 120 h post-transfection time points were tested.

### Quantitative reverse transcription polymerase chain reaction (qRT-PCR)

Total RNA was extracted with RNeasy kit (Qiagen, Valencia, CA, USA). RNA (1 μg) was reverse transcribed (GeneAmp RNA PCR, Applied Biosystems, Carlsbad, CA, USA) and amplified (Power SYBR Green, Applied Biosystems, Carlsbad, CA, USA) according to manufacturer's instructions for the translation initiation factors' targets (as predicted with bioinformatics tools): eIF4E's - AGPAT1, DOCK4, ARAP1, SERPINI1, IL23A, PTPRC, EGR1, LSP1, eIF4B, CRYGS, TRIB2 (primers' sequences described in supplementary Table S1) eIF4GI's - FBXO32, GAB2, HOXB9, PIM1, BCL2, SESN2, ASS1, DDIT3, PPM1E.

For microRNAs' qRT-PCR-RNA was extracted from with TRI Reagent and was converted to cDNA using the Quanta reverse transcription kit (Quanta-bioscience) according to manufacturer's instructions. Briefly, RNA was polyadenylated with ATP by poly (A) polymerase and reverse transcribed using poly (T) adapter primer. MicroRNAs were detected using a mature DNA sequence as the specific forward primer and a 3′ universal reverse primer provided in the Quanta RT kit. Human small nucleolar RNA SNORD44 was amplified as an internal control. Amplification was performed using Power SYBR Green PCR Master Mix (Quanta-bioscience).

All microarray data have been deposited at the National Center for Biotechnology Information Gene Expression Omnibus public database (GEO; http://www.ncbi.nlm.nih.gov/geo/) under accession number GSE62821(http://www.ncbi.nlm.nih.gov/geo/query/acc.cgi?acc=GSE62821).

### Cell count with trypan blue

Total cell counts as well as the respective proportion of viable and dead cells were enumerated by Trypan blue dye exclusion using a hematocytometer and a phase-contrast microscope [40].

### Materials

Autophagosome formation inhibitor 3 methyladenine (3MA) (Sigma, St. Louis, MO) (dissolved in ddw) was used at final concentrations of 5 mM.

### Bioinformatics microarray data analysis

Affymetrix GeneChip^®^ Human Gene 1.0 ST arrays (Affymetrix) were used for gene expression analysis according to instruction manual (http://media.affymetrix.com/support/technical/datasheets/gene_1_0_st_datasheet.pdf).

Microarray expression profiles were extracted from raw CEL files using Partek Genomic Suite (Partek^®^ software, version 6.4 Copyright © 2009; Partek Inc., http://www.partek.com) [41]. Data were normalized and summarized with the robust multi-average algorithm to allow for data comparison across the different arrays [42] followed by one-way analysis of variance (ANOVA). Genes were identified as differentially expressed with a cut-off *p* < 0.05 and 1.25 fold expression difference. Gene Ontology functional classification, enriched transcription factors, enriched microRNA and pathway enrichment analysis of differentially expressed genes was assessed with: ToppGene: http://toppgene.cchmc.org [43]; WebGestalt: http://bioinfo.vanderbilt.edu/webgestalt/ [44]. Venn diagrams were performed using Venny (http://bioinfogp.cnb.csic.es/tools/venny/index.html). The KD experiments were conducted 5 separate times of which duplicates underwent Affymetrix analysis. Validation (qPCR, Immunoblot) was performed on products from all 5 experiments.

### Statistical analysis

Student's paired *t* tests were employed in analysis of differences between cohorts. An effect was considered significant when *P*-value was equal to or less than 0.05. Microarray data were applied with non-parametric Kolmogorov-Smirnov test to determine that the data distribution is not normal. Wilcoxon signed ranks test used to compare two related samples to assess whether their populations mean ranks differ. All experiments were conducted at least three separate times.

## SUPPLEMENTARY FIGURE AND TABLE


