# Supplementary material for: eIF4E and eIF4GI have distinct and differential imprints on multiple myeloma's proteome and signaling
Source: Oncotarget. 2015 Jan 29;6(6):4315–29. doi: 10.18632/oncotarget.3008 (PMC4414192; doi:10.18632/oncotarget.3008)
Supplement: Supplementary file 1 [file oncotarget-06-4315-s001.pdf]

SUPPLEMENTARY FIGURE AND TABLE

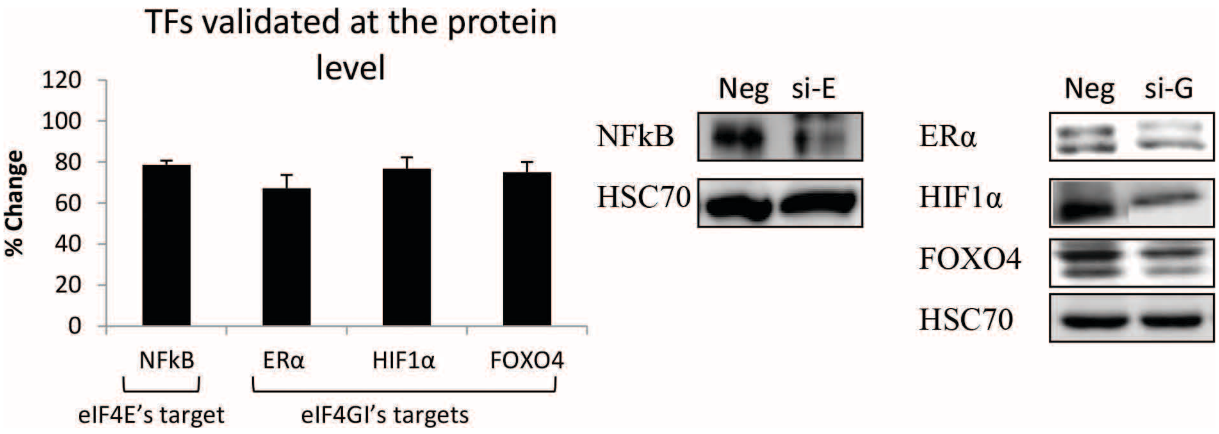

Supplementary Figure 1: Knockdown of eIF4E/eIF4GI downregulated their established targets (TFs): 8226 cells were transfected with negative siRNA control (Neg) or anti-eIF4E (si-E) or anti-eIF4GI (si-G) siRNA. Cells' protein from 96 h post-transfection experiments were immunobloted for eIF4E/eIF4GI established targets. Representative immunoblots and graphic presentations are presented (mean ± SE, $n = 6$). Protein quantities were normalized to HSC70 that served as a loading control. Assayed protein amounts measured in siRNA eIF4E/eIF4GI transfected cells were compared and normalized to negative siRNA transfected cells. Statistically significant differences ($*p < 0.05$) are depicted. These results were partly published in our previous publications.

Supplementary Table S1: eIF4E's & eIF4GI's TFs' targets

eIF4E's targets:

AGPAT1	Forward: 5-ACACCCCGACAGAGACAGAG-3
	Reverse: 5-CCTGGCCACAAATCCATTC-3-3
DOCK4	Forward: 5-GGATACCTACGGAGCACGAG-3
	Reverse: 5-AGCCATCACACTTCTCCAGG-3
ARAP1	Forward: 5-GCTTCCACGATCGCTACTTC-3
	Reverse: 5-ATAGGCCACTCCTTCTCAGG-3
SERPINI1	Forward: 5-AATTGAGGACGAGGTGGATG-3
	Reverse: 5-CCCCTGTAGCCATACTTTGC-3
IL23A	Forward: 5-CTCAGTGCCAGCAGCTTTC-3
	Reverse: 5-CCACACTGGATATGGGGAAC-3
PTPRC	Forward: 5-ACTCTTGGCATTTGGCTTTG-3
	Reverse: 5-CACTGGGCATCTTTGCTGTA
EGR1	Forward: 5-AGCCCTACGAGCACCTGAC-3
	Reverse: 5-GGAAAAGCGGCCAGTATAGG-3
LSP1	Forward: 5-CCAGACTACAGGCTGATGGC-3
	Reverse: 5-TGCCTGTCTCTCTCATGCTG-3
eIF4B	Forward: 5-TTTCCCTCTCCCAACATGG-3
	Reverse: 5-GTGCTTCCTCCACCAGTACC-3

(*Continued*)

**eIF4E's targets:**	
CRYGS	Forward: 5-CTTTCAGCACTGGGAAAACC-3
	Reverse: 5-AATCTGCACAGTCGCAATCA-3
TRIB2	Forward: 5-GTTCTACCAGATTGCCTCGG-3
	Reverse: 5-GAATGTAGGCGTCTTCCAGG-3
**eIF4GI's targets:**	
FBXO32	Forward: 5-GGGAAGCTTTCAACAGACTGG-3
	Reverse: 5-TCAGGGATGTGAGCTGTGAC-3
GAB2	Forward: 5-CTCCCGAGAAGAAGTTGAGG-3
	Reverse: 5-AGTTCAGGTTGATGATCCGC-3
HOXB9	Forward: 5-TAATCAAAGACCCGGCTACG-3
	Reverse: 5-GTGTAGGGACAGCGCTTTTT-3
PIM1	Forward: 5-CTCAAGCTCATCGACTTCGG-3
	Reverse: 5-ATGGTAGCGGATCCACTCTG-3
BCL2	Forward: 5-GACTGAGTACCTGAACCGGC-3
	Reverse: 5-CAGCCAGGAGAAATCAAACAG-3
SESN2	Forward: 5-CGCAGAGCTCAAGGACTACC-3
	Reverse: 5-CTCCACGGGGATGAAGG-3
ASS1	Forward: 5-CATTGGAATGAAGTCCCGAG-3
	Reverse: 5-CCAGGCCTTGTTTGATTTTG-3
DDIT3	Forward: 5-CCAAAATCAGAGCTGGAACC-3
	Reverse: 5-CCATCTCTGCAGTTGGATCA-3
PPM1E	Forward: 5-CCAAAATCAGAGCTGGAACC-3
	Reverse: 5-CCATCTCTGCAGTTGGATCA-3
